# Interaction of Nevirapine with the Peptide Binding Groove of HLA-DRB1*01:01 and Its Effect on the Conformation of HLA-Peptide Complex

**DOI:** 10.3390/ijms19061660

**Published:** 2018-06-04

**Authors:** Makoto Hirasawa, Katsunobu Hagihara, Koji Abe, Osamu Ando, Noriaki Hirayama

**Affiliations:** 1 Drug Metabolism & Pharmacokinetics Research Laboratories, Daiichi Sankyo Co., Ltd., 1-2-58 Hiromachi, Shinagawa-ku, Tokyo 140-8710, Japan; abe.koji.ce@daiichisankyo.co.jp (K.A.); ando.osamu.jy@daiichisankyo.co.jp (O.A.); 2 Biomarker Department, Daiichi Sankyo Co., Ltd., 1-2-58 Hiromachi, Shinagawa-ku, Tokyo 140-8710, Japan; hagihara.katsunobu.fc@daiichisankyo.co.jp; 3 Institute of Advanced Biosciences, Tokai University, 4-1-1 Kitakaname, Hiratsuka-shi, Kanagawa 259-1292, Japan; hirayama@is.icc.u-tokai.ac.jp

**Keywords:** nevirapine, HLA (human leukocyte antigen), hepatic hypersensitivity reaction, idiosyncratic drug toxicity, MD (molecular dynamics) simulation

## Abstract

Human leukocyte antigen (HLA)-DRB1*01:01 has been shown to be involved in nevirapine-induced hepatic hypersensitivity reactions. In the present study, in silico docking simulations and molecular dynamics simulations were performed to predict the interaction mode of nevirapine with the peptide binding groove of HLA-DRB1*01:01 and its possible effect on the position and orientation of the ligand peptide derived from hemagglutinin (HA). In silico analyses suggested that nevirapine interacts with HLA-DRB1*01:01 around the P4 pocket within the peptide binding groove and the HA peptide stably binds on top of nevirapine at the groove. The analyses also showed that binding of nevirapine at the groove will significantly change the inter-helical distances of the groove. An in vitro competitive assay showed that nevirapine (1000 μM) increases the binding of the HA peptide to HLA-DRB1*01:01 in an allele-specific manner. These results indicate that nevirapine might interact directly with the P4 pocket and modifies its structure, which could change the orientation of loaded peptides and the conformation of HLA-DRB1*01:01; these changes could be distinctively recognized by T-cell receptors. Through this molecular mechanism, nevirapine might stimulate the immune system, resulting in hepatic hypersensitivity reactions.

## 1. Introduction

Nevirapine (Viramune^®^) is an orally available antiretroviral agent, which inhibits reverse transcriptase. It is used in combination with other antiretroviral agents for the treatment of HIV/AIDS infections and is included in the WHO Model List of Essential Medicines. Although it is generally well-tolerated and effective, it has been associated with hypersensitivity reactions (HSRs) in approximately 5% of patients with various clinical phenotypes, including cutaneous, hepatic, and systemic symptoms [1,2,3,4]. Several features of nevirapine HSRs, including the delayed onset of reaction, more rapid and severe development of symptoms after rechallenge, and association with the percentage of pretreatment CD4 T-cells, suggest that nevirapine HSRs are immune-mediated [3,5].

Various genetic association studies have shown that cytochrome P450 (CYP) 2B6 genotypes and human leukocyte antigen (HLA) alleles are predisposed to the development of nevirapine HSRs [4,5,6,7,8]; however, these associations were very complicated. For example, CYP2B6 G516T polymorphism increased nevirapine plasma exposure [9] and was associated with its cutaneous HSRs [6], but not with hepatic ones [6,10]. In addition, unlike the cases of HLA-B*57:01 in abacavir HSRs [11] and HLA-B*58:01 in allopurinol-induced Stevens-Johnson syndrome (SJS) and toxic epidermal necrolysis (TEN) [12], multiple class I and class II HLA alleles, which have variable distribution and risk across ethnic groups, have been associated with nevirapine HSRs [6,13,14]. Interestingly, associations with protective HLA alleles have also been reported [7,13,15]. Furthermore, the associations with predisposing HLA alleles tend to be phenotype-specific [6,14,16], where HLA-C*04 has been associated with cutaneous HSRs, in particular SJS/TEN [6,7,8,13,14], but HLA-DRB1*01 has been associated with hepatic HSRs. The exclusion of individuals with coincident cutaneous and hepatic HSRs resulted in a significant increase of the odds ratios [5,6]. These results suggest that cutaneous and hepatic HSRs might have fundamentally different mechanisms.

**Figure 1:**
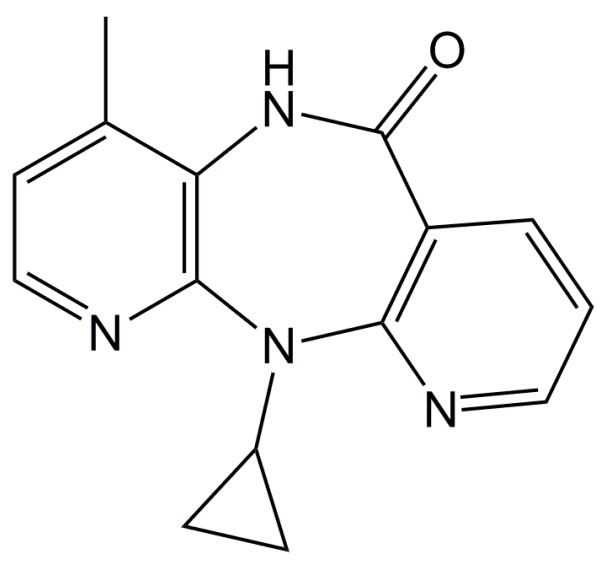
Chemical structure of nevirapine.

We previously reported in silico and in vitro interactions of lapatinib [17] and ximelagatran [18] with HLA-DRB1*07:01, which predisposed to the development of idiosyncratic hepatotoxicity by both drugs, and indicated that these interactions might trigger immune responses, leading to drug-related toxicity. In the present study, we performed in silico docking simulations to predict the interaction mode of nevirapine (Figure 1) with the peptide binding groove of HLA-DRB1*01:01, as well as molecular dynamics (MD) simulations to predict the possible effect of nevirapine on the structure of HLA-DRB1*01:01 and the position and orientation of the bound peptide derived from hemagglutinin (HA). An in vitro competitive assay was performed to evaluate the effect of nevirapine on the binding of ligand peptides to three HLA-DR alleles.

## 2. Results

### 2.1. Docking Simulations

**Figure 2:**
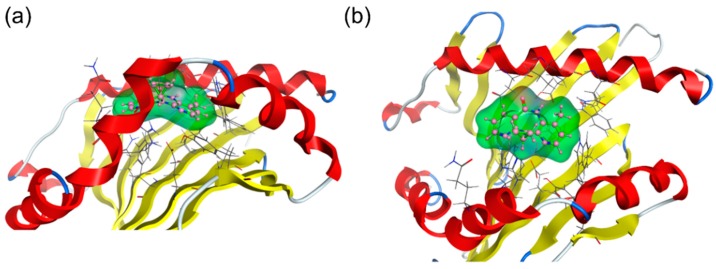
Binding mode of nevirapine to human leukocyte antigen (HLA)-DRB1*01:01 in docking simulations, (**a**) side view and (**b**) top view. The structures of HLA-DRB1*01:01, nevirapine, and amino acid residues of HLA-DRB1*01:01 in the vicinity of nevirapine are depicted in cartoon mode, ball-and-stick model, and wire model, respectively.

In silico docking simulations were performed to predict the interaction mode of nevirapine with the peptide binding groove of HLA-DRB1*01:01. The binding affinity of nevirapine to HLA-DRB1*01:01 was judged by a scoring function of generalized-Born volume integral/weighted surface area (GBVI/WSA_dG). The lowest GBVI/WSA_dG value of the complex between nevirapine and HLA-DRB1*01:01 was −5.18 kcal/mol. This value is higher than that of the complex between HLA-B*14:02 and nevirapine (−6.40 kcal/mol) simulated by the same software system [19], indicating that the interaction of nevirapine with HLA-DRB1*01:01 was weaker than that with HLA-B*14:02. The binding mode of nevirapine at the peptide binding groove of HLA-DRB1*01:01 with the lowest GBVI/WSA_dG value is shown in Figure 2. Nevirapine sprawls out across the pocket P4–P6.

### 2.2. Molecular Dynamics (MD) Simulations

**Figure 3:**
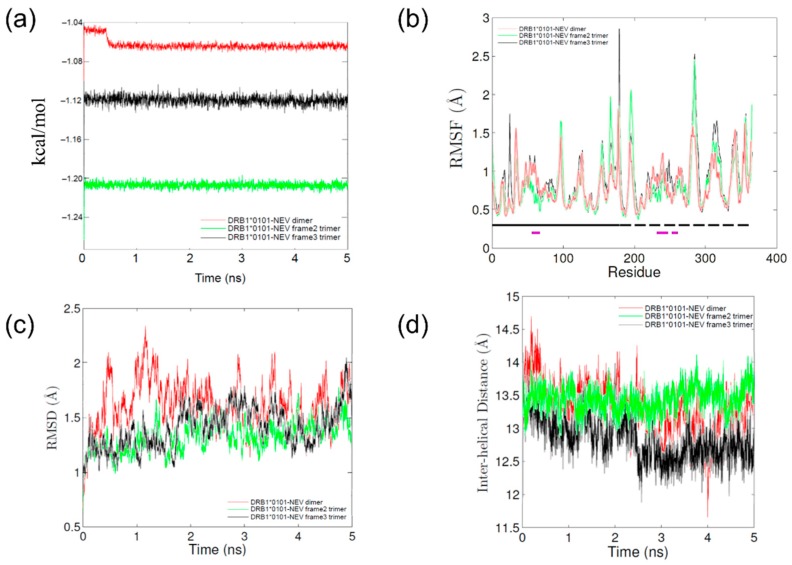
Parameter profiles of molecular dynamics (MD) simulations of nevirapine-bound HLA-DRB1*01:01. (**a**) Calculated energies vs. time plot; (**b**) RMSF values of polypeptide backbone. The location of α and β chains and residues that comprise the peptide binding groove helices are indicated by the solid and dashed lines running just above the *x*-axis, respectively. The α chain (**solid black**), α chain helix (**solid purple**), β chain (**dashed black**), and β chain helix (**dashed purple**); (**c**) Root mean square deviation (RMSD) values of polypeptide backbone vs. time plot; and (**d**) The average distance between each Cα in the helix of the α chain and the closest Cα atom in the helix of the β chain.

MD simulations were performed to evaluate the possible effect of nevirapine on the structure of the peptide binding groove of HLA-DRB1*01:01 and the position and orientation of bound HA peptide. Each of the total energies of MD simulations stabilized within approximately 0.5 ns (Figure 3a) indicating that the simulations were energetically stable. The flexibility of HLA-DRB1*01:01 molecule was very similar in all simulations (Figure 3b). As shown in Figure 3c, the root mean square deviation (RMSD) values stabilized after 1–2 ns in all simulations, which indicated that the systems relaxed quickly.

The interaction of nevirapine with HLA-DRB1*01:01 resulted in the closing motion of the peptide binding groove (Figure 3d, Table 1), which differs from the tightly closed binding groove observed with lapatinib-DRB1*07:01 [17] and the open groove observed with lapatinib-DRB1*01:01, lapatinib-DRB1*15:01, [17] and ximelagatran-DRB1*07:01 [18]. The mean inter-helical distance of the peptide binding groove of HLA-DRB1*01:01 in the presence of nevirapine (13.1 ± 0.4 Å; Table 1) was very similar to that observed in apo simulation (13.0 ± 0.4 Å), which reflected the transition of HLA-DR molecules from a peptide-receptive conformation to a non-receptive one in the absence of any ligand molecules [20]. This is reasonable considering the much smaller molecular size of nevirapine than that of lapatinib and ximelagatran.

**Figure 4:**
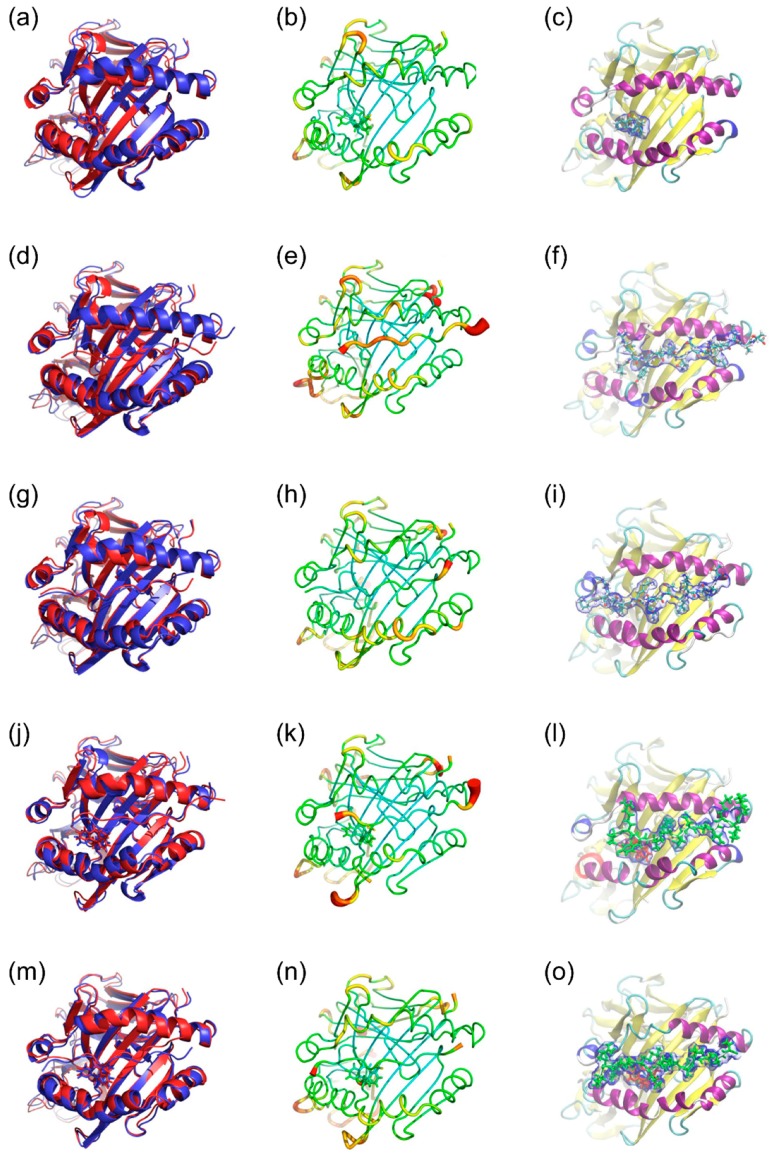
Simulated representative structures of nevirapine-bound HLA-DRB1*01:01 (**a**–**c**), hemagglutinin (HA) peptide-bound HLA-DRB1*01:01 in frame 2 (**d**–**f**) and frame 3 (**g**–**i**), and nevirapine-bound HLA-DRB1*01:01-HA peptide in frame 2 (**j**–**l**) and frame 3 (**m**–**o**). (**a**,**d**,**g**,**j**,**m**): Alignment of initial structure (**red**) and representative structure (**blue**); (**b**,**e**,**h**,**k**,**n**): Sausage plot of the structure, where the color and thickness of HLA-DRB1*01:01 are proportional to the RMSF of Cα; and (**c**,**f**,**i**,**l**,**o**): The volume occupied by nevirapine and HA peptide. The blue envelope outlines the 50% occupancy volume of nevirapine (**red**) and HA peptide (**green**).

Figure 4 shows structural analyses of five sets of MD simulations presented in three panels for each. In the absence of HA peptide, nevirapine bound to HLA-DRB1*01:01 in or near the P4 pocket in a stable conformation (Figure 4c) and had a small impact on the overall configuration of the α and β chains of HLA-DRB1*01:01, compared to that of lapatinib [17]. Regarding HA peptide-bound simulations, three starting frames (2, 3, and 4) were considered, and frame 3 was the most likely because it was stable and exhibited good contacts with both sides of the groove. The conformation was the closest to the X-ray crystal structure (Figure 4d–f). Frame 2 was also considered for the trimer (nevirapine, HLA-DRB1*01:01, and HA peptide) simulations because of its total energy, although the binding mode and closing motion of the peptide binding groove were somehow unusual (Figure 4g–i, Table 1). In the trimer simulations, conformational changes of both nevirapine and HA peptide during 5-ns simulations were slight in both frames. Nevirapine seems to interact mainly with the peptide binding groove around the P4 pocket, whereas HA peptide bound on top of nevirapine. Slight conformational changes were observed in the α and β chains of HLA-DRB1*01:01 with the α chain helix being maintained. Considering the high root mean square fluctuation (RMSF) values of HA peptide ends in frame 2, the HA peptide in frame 3 appears more stably bound to HLA-DRB1*01:01 in the trimer simulations.

**Figure 5:**
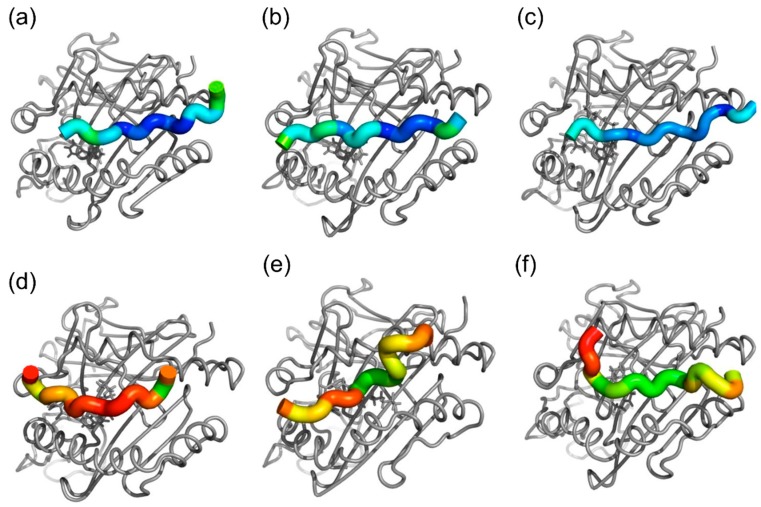
Drug-induced peptide displacements in (**a**) nevirapine-bound HLA-DRB1*01:01-HA peptide in frame 2 and (**b**) frame 3; (**c**) lapatinib-bound HLA-DRB1*01:01-HA peptide in frame 2 (**d**) and frame 3; (**e**) lapatinib-bound HLA-DRB1*07:01-peptide from tetanus toxoid (TT) in frame 3; and (**f**) ximelagatran-bound HLA-DRB1*07:01-TT peptide in frame 3. Each residue in the ligand peptides is colored according to the displacement of the Cα in the trimer simulations, relative to its position in the HLA-peptide dimer simulations. The same color scale is used and the range is from 0 (**blue**) to 10 Å (**red**). Small values mean that the drug has little effect on the position of ligand peptides in the binding groove. Drugs are shown using grey stick representation.

Figure 5 shows the differences in the positions and orientations of ligand peptides between representative structures of trimer and HLA-peptide dimer simulations. Nevirapine had much smaller effects on the position and orientation of the ligand peptide than lapatinib and ximelagatran.

### 2.3. HLA Class II Competitive Assay

To evaluate the effect of nevirapine on the binding of ligand peptides to three HLA-DR molecules, a DELFIA-based competitive assay was conducted. Nevirapine increased the binding of HA peptide to soluble HLA-DRB1*01:01 molecule only at 1000 μM (*p* < 0.0001), whereas there were no significant or concentration-dependent effects on the binding of the peptide derived from tetanus toxoid (TT) to HLA-DRB1*07:01 and that derived from myelin basic protein (MBP) to HLA-DRB1*15:01 (Table 2).

The concentration of nevirapine that possibly bound to HLA-DR molecules in the competitive assay sample, where nominally 1000 μM of nevirapine was applied, was measured by liquid chromatography-tandem mass spectrometry (LC-MS/MS). However, a much higher concentration than the theoretically highest one (4.7 nM) with a large variance was detected in the absence of HLA-DR molecules, which indicated high non-specific binding of nevirapine to the plastic tubes and plates in the absence of HLA-DR molecules. In addition, the nevirapine concentration in the presence of both the ligand peptide and HLA-DR was lower than that in the absence of the ligand peptide for all three HLA-DR alleles, which indicated that the presence of the ligand peptide also attenuated the non-specific binding of nevirapine to tubes and plates. Taken together, the nevirapine bound to HLA-DR molecules could not be detected in this experiment owing to its high non-specific binding.

## 3. Discussion

Associations of multiple phenotypes of nevirapine HSRs with various HLA class I and class II alleles across several ethnic groups have been reported [4,5,6,7,8,13,14,16]. Among the multiple HLA alleles associated with nevirapine HSRs, HLA-C*04:01 is most often associated and commonly carried across ethnic groups [13]. Several docking studies recently have shown the binding of nevirapine in the B and/or F pockets [8,13] within the peptide binding groove of HLA-C*04:01. Pavlos et al. [13] reported that HLA-C risk alleles for nevirapine cutaneous HSRs (*04:01, *05:01, and *18:01) shared the unique F pocket motif, as well as Arg156. They proposed that the disease-causing peptides were anchored in the F pocket together with nevirapine and stabilized by Arg156 in the central portion (P3-P5-P6), which could propagate T-cell mediated responses. In addition, they reported that the P4 pocket motif was also shared by HLA-DRB1 risk alleles (*01:(01/02/03) and *04:(04/05/08/10)).

Present in silico studies have shown that nevirapine sprawls out across the P4–P6 pockets in the docking simulations (Figure 2) and binds to the P4 pocket in a stable conformation both in the presence and absence of HA peptide in the MD simulations (Figure 4). These findings are in accordance with the reported predisposing effect of the shared P4 pocket motif. It is noteworthy that the present docking simulations indicated that the interaction of nevirapine with the peptide binding groove of HLA-DRB1*01:01 was weaker than that of other idiosyncratic drug toxicity-causing drugs, such as ximelagatran [18] and allopurinol [21].

Moreover, Pavlos et al. reported that nevirapine did not affect the repertoire of peptides presented on HLA-DRB1*01:01 in L2 cells, and any peptide eluted from nevirapine-treated cells did not show a significantly higher binding affinity to HLA-DRB1*01:01 in the presence of nevirapine than in the absence of it. These results were unexpected considering the relatively small molecular size and the simulated direct interaction mode of nevirapine with the P4 pocket of HLA-DRB1*01:01, which indicates the likelihood of the “altered-repertoire” mechanism of nevirapine-induced immune stimulation, similar to abacavir hypersensitivity [22].

However, in the present in vitro competitive assay, nevirapine increased the binding of HA peptide to HLA-DRB1*01:01 in an allele-specific manner at 1000 μM (Table 2), although its concentration dependency was not clear, in comparison to that of lapatinib, which increased the binding of the TT peptide to HLA-DRB1*07:01 in a concentration-dependent manner [17]. The lack of effect at lower concentrations of nevirapine might be consistent with its relatively low affinity to HLA-DRB1*01:01, as shown in the docking simulations. This peculiar concentration dependency might account for the negative results of the elution studies conducted by Pavlos et al. [13], where nominally 100 μg/mL (375.5 μM) of nevirapine was applied to the cells.

Additionally, nevirapine might not influence the binding affinity of the ligand peptides to HLA-DRB1*01:01 but could appreciably affect their conformation and orientation at lower concentrations, which are undetectable in the present competitive assay and probably in the reported elution studies. This hypothesis is supported by the present MD simulation results, which suggested that HA peptide can bind to HLA-DRB1*01:01 on top of nevirapine bound to the P4 pocket (Figure 4) but the effect of nevirapine on the position and orientation of the HA peptide on HLA-DRB1*01:01 was much less than those of ximelagatran and lapatinib (Figure 5). This indicates that nevirapine might neither inhibit nor enhance the binding of the ligand peptides to HLA-DRB1*01:01 robustly, which is unlike the results observed with HLA-DRB1*07:01-ximelagatran [18] and HLA-DRB1*07:01-lapatinib [17].

The binding mode of HA peptide in the trimer simulations was seemingly contradictory to the results of molecular modeling reported by Pavlos et al. [13], where the leucine residue of the sequence logo peptide (YRSLKAQRV), which was identified from peptides bound to HLA-DRB1*01:01 in nevirapine-treated cells, occupied the P4 pocket, and thus nevirapine was considered unlikely to bind directly to HLA-DRB1*01:01 in the presence of the peptide. However, in the present MD simulations (frame 3 trimer), P1–P5 (Tyr-Val-Lys-Gln-Asn) moved slightly to cover nevirapine with Tyr and Lys, having a non-bonded interaction with nevirapine. Additionally, the α and β helices slightly changed their conformations to accommodate the binding of the HA peptide. This finding showed the advantage of MD simulations in predicting the possible effects of small-molecule drugs on the position and orientation of the ligand peptides in the binding groove of HLA class II molecules.

As shown in Figure 5, the degree of nevirapine-induced changes in the position and orientation of the HA peptide in HLA-DRB1*01:01 was much smaller than that of lapatinib- and ximelagatran-induced changes in the position of the TT peptide in HLA-DRB1*07:01. The relevant small conformational change in the HLA-peptide complex was considered to be insufficient to trigger the immune response. However, the mechanism of immune stimulation induced by small structural changes in the surface of HLA-ligand complexes was clearly shown for chronic beryllium disease (CBD) associated with HLA-DP2 [23]. Petukh et al. [24] analyzed the conformational changes in HLA-DP2 molecule induced by the binding of the ligand peptides in the presence and absence of Be^2+^ cation by comparing the inter-helical distances in HLA-DP2 using MD simulations. The simulations indicated that the conformational changes induced by the binding of the ligand peptides in the presence of Be^2+^ were distinctly different from those caused by natural peptides, which could trigger immune responses in the absence of Be^2+^, and these conformational changes were expected to trigger a completely different signaling cascade of immune reactions, resulting in CBD.

In the present MD simulations, nevirapine induced significant differences in the mean inter-helical distances of the peptide binding groove of HLA-DRB1*01:01 (Table 1). Accordingly, the interaction of nevirapine with HLA-DRB1*01:01 might induce conformational changes not only in the ligand peptide but also in HLA-DRB1*01:01. These changes might be recognized differently by T cell receptors (TCRs) and trigger nevirapine-induced hepatic HSRs.

## 4. Materials and Methods

### 4.1. Docking Simulations

A crystal structure of HLA-DRB1*01:01 (PDB ID: 3PDO) deposited at the Protein Data Bank [25] was used in this study. The binding mode and affinity of nevirapine to the peptide binding groove of HLA-DRB1*01:01 were determined by docking simulations using the program ASEDock [26]. The scoring function of GBVI/WSA_dG [27] was used to evaluate the binding affinity. The software system molecular operating environment (MOE) [28] was used.

### 4.2. MD Simulations

A crystal structure of HLA-DRB1*01:01 (PDB ID: 1AQD) was used in this study. The top scoring mode of nevirapine with HLA-DRB1*01:01 in the docking simulations performed using AutoDock Vina [29] was selected as a starting conformation for MD simulation. HA peptide (HA 306–318; PRYVKQNTLKLAT) was used as a ligand peptide for HLA-DRB1*01:01. The structure of the HA peptide was generated by homology modeling using a template structure (PDB ID: 1DLH, chain C; PKYVKQNTLKLAT). To select the correct peptide registration, we conducted a short translational scan by sliding the HA peptide along the axis of the binding groove and running additional MD simulations. After modeling a series of peptide registration frames, frame 2 and frame 3 were chosen as starting points for MD simulations, considering the energetic stability, contact with α and β chains, and EpiMatrix-predicted registration. MD simulations were carried out as previously described [17]. Briefly, each system was protonated at pH 7.4 and then solvated in a periodic rectangular box of TIP3P water with 10-Å padding between the edge of the box and the nearest solute atom. Sodium ions were added to neutralize the system. The system was relaxed and then gradually heated to 300 K. Production simulations were performed in the canonical constant-temperature, constant-volume (NVT) ensemble. An integration time step of 4 fs was used. The “representative structure” of each simulation was selected as the conformation, which was the most representative of the conformations assumed over the final 1000 frames of the simulations. MD simulations of nevirapine-bound HLA-DRB1*01:01 in the absence and presence of HA peptide lasted 5 ns for each.

### 4.3. HLA Class II Competitive Assay

The competitive assay was conducted as previously reported with minor modifications [30]. Briefly, soluble HLA-DR molecules (20 nM), provided by Benaroya Research Institute (Seattle, WA, USA), were incubated in quadruplicate with nevirapine at concentrations ranging between 8 and 1000 μM and biotinylated control ligand peptides (HA 306–318: PRYVKQNTLKLAT for HLA-DRB1*01:01, TT 830–844: QYIKANSKFIGITEL for HLA-DRB1*07:01, and MBP 94–112: NPVVHFFKNIVTPRTPPPS for HLA-DRB1*15:01) at concentrations lower than their Michaelis-Menten constants for 48 h at 37 °C to reach equilibrium. HLA-peptide complexes were then captured onto 96-well plates coated with L243 anti-HLA-DR antibody (BioLegend, San Diego, CA, USA). The plates were washed and incubated with Europium-labeled streptavidin (Perkin-Elmer, Waltham, MA, USA) for 1 h at room temperature. The plates were washed again, and DELFIA enhancement solution (Perkin-Elmer) was added for 20 min at room temperature to develop the plates before they were read using an EnVision plate reader. An aliquot of the incubation sample containing 1000 μM of nevirapine was also captured onto other L243-coated plates. The plates were washed, and nevirapine bound to HLA-DR molecules was extracted with 150 μL of acetonitrile. The concentrations of nevirapine in the extracted samples were determined by LC-MS/MS using a Shim-pack XR-ODS (30 × 2 mm i.d., 2.2 μm) column and Prominence LC-20A system (Shimadzu Corp., Kyoto, Japan) coupled with a Sciex API 4000 triple quadrupole mass spectrometer (Applied Biosystems, Foster City, CA, USA). The aqueous mobile phase (A) was H_2_O/100 mM CH_3_CO_2_NH_4_/CH_3_CN (900/50/50, *v*/*v*/*v*), and the organic mobile phase (B) was CH_3_CN/100 mM CH_3_CO_2_NH_4_ (1000/50, *v*/*v*). Gradient elution was used as follows: 50% B for the first 0.5 min, increased to 100% B from 0.5 to 1 min, and maintained at 100% B for additional 0.75 min. The total run time was 1.75 min at a flow rate of 0.75 mL/min. Ionization was conducted in the positive ion mode using the transition *m*/*z* 267.1 to *m*/*z* 226.1. The retention time of nevirapine was 0.96 min. The lower and upper limits of quantification of the assay were 0.03 and 300 nM, respectively.

### 4.4. Statistical Analysis

A two-tailed Dunnett’s test was carried out using SAS System Release 9.2 (SAS Institute Inc., Cary, NC, USA) to compare the relative fluorescence counts (% of dimethyl sulfoxide control for each HLA-DR allele) in the presence and absence of nevirapine. A *p*-value < 0.05 was considered statistically significant.

## 5. Conclusions

The present study suggested that nevirapine might directly interact with the peptide binding groove of HLA-DRB1*01:01 around the P4 pocket, which could induce conformational changes in the ligand peptide and HLA-DRB1*01:01. These conformational changes recognized by TCR differently from the natural peptide-HLA-DRB1*01:01 complex would stimulate the immune system and trigger nevirapine-induced hepatic HSRs.

## Figures and Tables

**Table 1:** Mean molecular dynamics (MD) simulations parameters.

Hemagglutinin Peptide	Nevirapine	Energies (kcal/mol)	RMSF (Å)	Inter-Helical Distance (Å)	Slope of Inter-Helical Distance (Å/ns)
-	−	−106715 ± 309.9	1.0 ± 0.4	13.0 ± 0.4	−0.12
-	+	−106370 ± 533.5	0.8 ± 0.3	13.1 ± 0.4	−0.14
Frame 2	−	−111392 ± 579.8	0.9 ± 0.4	12.7 ± 0.2	−0.16
Frame 3	−	−106078 ± 323.1	0.9 ± 0.3	14.2 ± 0.3	0.04
Frame 2	+	−120550 ± 243.6	0.8 ± 0.3	13.5 ± 0.2	0.02
Frame 3	+	−112166 ± 230.5	0.9 ± 0.3	12.6 ± 0.2	−0.13

The total energies, average root mean square fluctuation (RMSF), distance between the α-helices flanking the binding groove, and slope of inter-helical distance curve are listed. Values represent the mean ± standard deviation (SD) during 5-ns simulations.

**Table 2:** The effect of nevirapine on the binding of ligand peptides to HLA-DR molecules.

HLA Allele		DRB1*01:01	DRB1*07:01	DRB1*15:01
Concentration of nevirapine (μM)	1000	263.9 ± 15.5 ^#^	62.6 ± 3.9	101.3 ± 14.9
	200	99.7 ± 11.3	110.4 ± 5.5	94.6 ± 10.6
	40	106.2 ± 6.5	76.9 ± 4.6	93 ± 10.4
	8	101.9 ± 8.1	132.2 ± 64	98.9 ± 3.4

The effects of nevirapine on the binding of ligand peptides to three HLA-DRs are expressed as % binding of ligand peptides in the presence of nevirapine to that in dimethyl sulfoxide (DMSO) control (*n* = 8). Values represent the mean ± SD of quadruplicate. ^#^ *p* < 0.001, compared with the DMSO control.

## Abbreviations

HLA	human leukocyte antigen
HA	hemagglutinin
HSR	hypersensitivity reaction
CYP	cytochrome P450
SJS	Stevens-Johnson syndrome
TEN	toxic epidermal necrolysis
MD	molecular dynamics
GBVI/WSA_dG	generalized-Born volume integral/weighted surface area
RMSD	root mean square deviation
RMSF	root mean square fluctuation
SD	standard deviation
TT	tetanus toxoid
MBP	myelin basic protein
LC-MS/MS	liquid chromatography-tandem mass spectrometry
DMSO	dimethyl sulfoxide
CBD	chronic beryllium disease
TCR	T-cell receptor
